# Resistance to permethrin alters the gut microbiota of *Aedes aegypti*

**DOI:** 10.1038/s41598-021-93725-4

**Published:** 2021-07-13

**Authors:** Ephantus J. Muturi, Christopher Dunlap, Chelsea T. Smartt, Dongyoung Shin

**Affiliations:** 1 Crop Bioprotection Research Unit, USDA, Agricultural Research Service, National Center for Agricultural Utilization Research, 1815 N University St., Peoria, IL 61605 USA; 2 Florida Medical Entomology Laboratory, University of Florida, Vero Beach, FL 32962 USA

**Keywords:** Ecology, Microbiology

## Abstract

Insecticide resistance has emerged as a persistent threat to the fight against vector-borne diseases. We compared the gut microbiota of permethrin-selected (PS) strain of *Aedes aegypti* relative to the parent (KW) strain from Key West, Florida. Bacterial richness but not diversity was significantly higher in PS strain compared to KW strain. The two mosquito strains also differed in their gut microbial composition. *Cutibacterium* spp., *Corynebacterium* spp., *Citricoccus* spp., *Leucobacter* spp., *Acinetobacter* spp., *Dietzia* spp., and *Anaerococcus* spp. were more abundant in PS strain than in KW strain. In contrast, *Sphingomonas* spp., *Aquabacterium* spp., *Methylobacterium* spp., *Flavobacterium* spp., *Lactobacillus* spp., unclassified Burkholderiaceae and unclassified Nostocaceae were more abundant in KW strain compared to PS strain. PS strain was enriched with propionate metabolizers, selenate reducers, and xylan, chitin, and chlorophenol degraders while KW strain was enriched with sulfur oxidizers, sulfur metabolizers, sulfate reducers and naphthalene and aromatic hydrocarbons degraders. These findings demonstrate an association between the gut microbiota and insecticide resistance in an important vector species and sets the foundation for future studies to investigate the contribution of gut microbiota to evolution of insecticide resistance in disease vectors.

## Introduction

Suppression of vector populations using insecticides is a critical component of the global strategy to manage mosquito-borne diseases. This strategy utilizes a variety of chemical compounds that target both the larval and adult stages of the vectors. Unfortunately, the heavy reliance on insecticides has resulted in mosquito resistance to all classes of insecticides approved for vector control, threatening the future of insecticides in combating mosquito-borne diseases^1–4^. As a result, there is worldwide research attention on resistance mechanisms and management.

The best-known mechanisms of insecticide resistance are those associated with behavioral, genetic, and physiological changes in the mosquito, and are broadly divided into two major categories: behavioral and physiological resistance. In behavioral resistance, insects develop the ability to avoid or reduce exposure to insecticides through direct contact excitation (irritancy) or non-contact spatial repellency^5^. In contrast, physiological resistance is associated with the ability to survive exposure to lethal insecticide concentrations as a result of physiological modifications that lead to target site insensitivity, enhanced metabolic detoxification and/or reduced cuticular penetration^5,6^.

Mounting evidence also suggest that the microbial communities associated with insects may contribute to insecticide resistance but are rarely considered as a mechanism of insecticide resistance in disease vectors. The microbial composition of *An. albimanus* Wiedemann differed between fenitrothion-susceptible and -resistant strains but not between pyrethroid-resistance and -susceptible strains, although mosquitoes exposed to permethrin and cypermethrin had different microbial composition relative to non-exposed mosquitoes^7,8^. Temephos-resistant strain of *An. stephensi* Liston became susceptible when their bacterial communities were disrupted by adding antibiotics to the larval rearing environment^9^. The gut microbial community of the German cockroach, *Blatella germanica* L., differed between indoxacarb-resistant and susceptible laboratory strains, and treatment of an indoxacarb-resistant laboratory strain with doxycycline increased susceptibility to orally administered indoxacarb insecticide^10^. Additionally, the susceptible strain became more resistant to indoxacarb when fed on gut microbes from resistant strain via fecal transplant^10^.

Similar findings have been reported in agricultural pests where most studies on the link between insect-associated bacteria and insecticide resistance have been conducted. The gut symbiont *Citrobacter* sp. was shown to mediate resistance to the organophosphate insecticide trichlorfon in the oriental fruit fly *Bactrocera dorsalis* Hendel^11^. In the diamondback moth, *Plutella xylostella* L., the gut microbiota differed significantly between chlorpyrifos- and fipronil-susceptible and -resistant strains^12^. Further studies revealed that *Enterococcus* spp. isolated from the guts of *P. xylostella* enhanced resistance to chlorpyrifos while *Serratia* spp. decreased resistance to chlorpyrifos^13^. The presence of *Rickettsia* spp. enhanced susceptibility of the sweet potato whitefly *Bemisia tabaci* to acetamiprid, thiamethoxam, spiromesifen and pyriproxyfen but had no effect on imidacloprid and diafenthiuron treatment^14^. Follow-up studies revealed a strong positive association between *Rickettsia* densities and susceptibility to insecticides^15^. Elimination of gut bacteria through oral administration of antibiotics reduced susceptibility of *Vanessa cardui* (L.), *Manduca sexta* (L.), *Pieris rapae* (L.), *Heliothis virescens* (F.) and *Lymantria dispar* (L.) to *Bacillus thuringiensis* (Bt) suggesting that indigenous gut bacteria contribute to Bt susceptibility. The stinkbug *Riptortus pedestris* was also shown to acquire fenitrothion-degrading *Burkholderia* strains from the soil leading to host resistance to fenitrothion^16^. Collectively, these studies suggest that microbe-mediated insecticide resistance and susceptibility may be widespread in nature and should be considered in studies that investigate the mechanisms of insecticide resistance.

In this study, we compared the gut microbiota of permethrin-selected strain of the yellow fever mosquito *Aedes aegypti* (L.) relative to its parent strain collected from Key West, Florida. *Aedes aegypti* is the primary vector of dengue, chikungunya, yellow fever, and Zika viruses and its control has recently been complicated by the rapid spread of insecticide resistance^17–19^. Thus, studies that improve current understanding of insecticide resistance mechanisms are urgently needed to facilitate the development of novel approaches for resistance prevention and management. The findings of this study provide the baseline for further studies on microbe-mediated insecticide resistance in mosquitoes and other arthropod vectors.

## Results

### Bacterial community composition

A total of 388,561 reads were obtained from 60 samples (n = 30/mosquito strain). The number of reads per sample ranged from 0 for sample number KW7 to 42,989 for sample number PS20 (mean = 6477.52 ± 926.02). After excluding samples with less than 1218 sequences and OTUs with less than 10 sequences, a total of 50 samples were retained for downstream analyses. Analysis based on the 50 samples resulted in a total of 1152 OTUs belonging to 23 bacterial phyla, 193 families and 385 genera.

**Figure 1:**
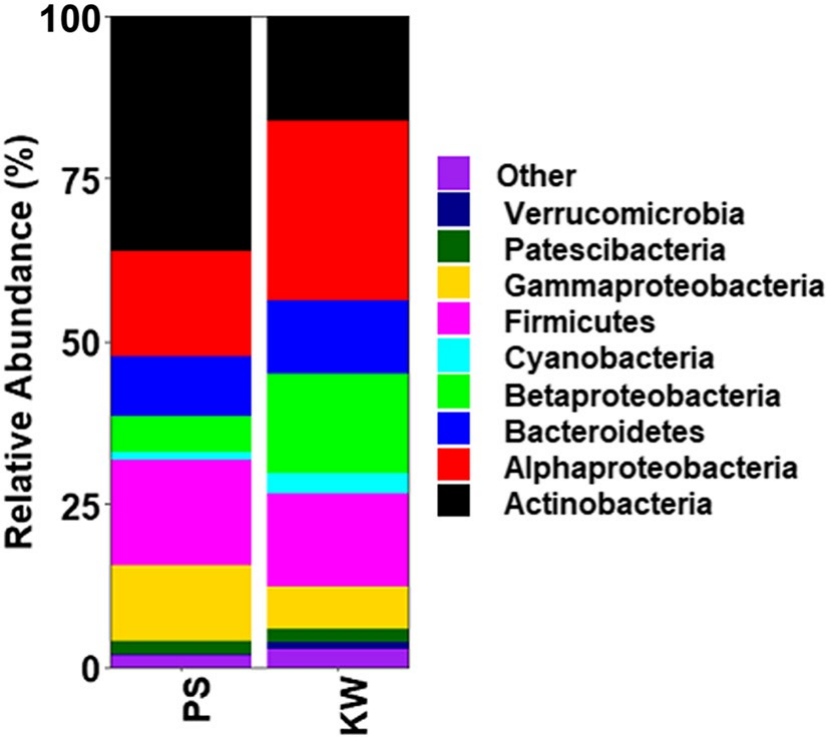
Relative abundances of bacterial phyla associated with permethrin-selected (PS) and parent strain (KW) of *Aedes aegypti*.

**Figure 2:**
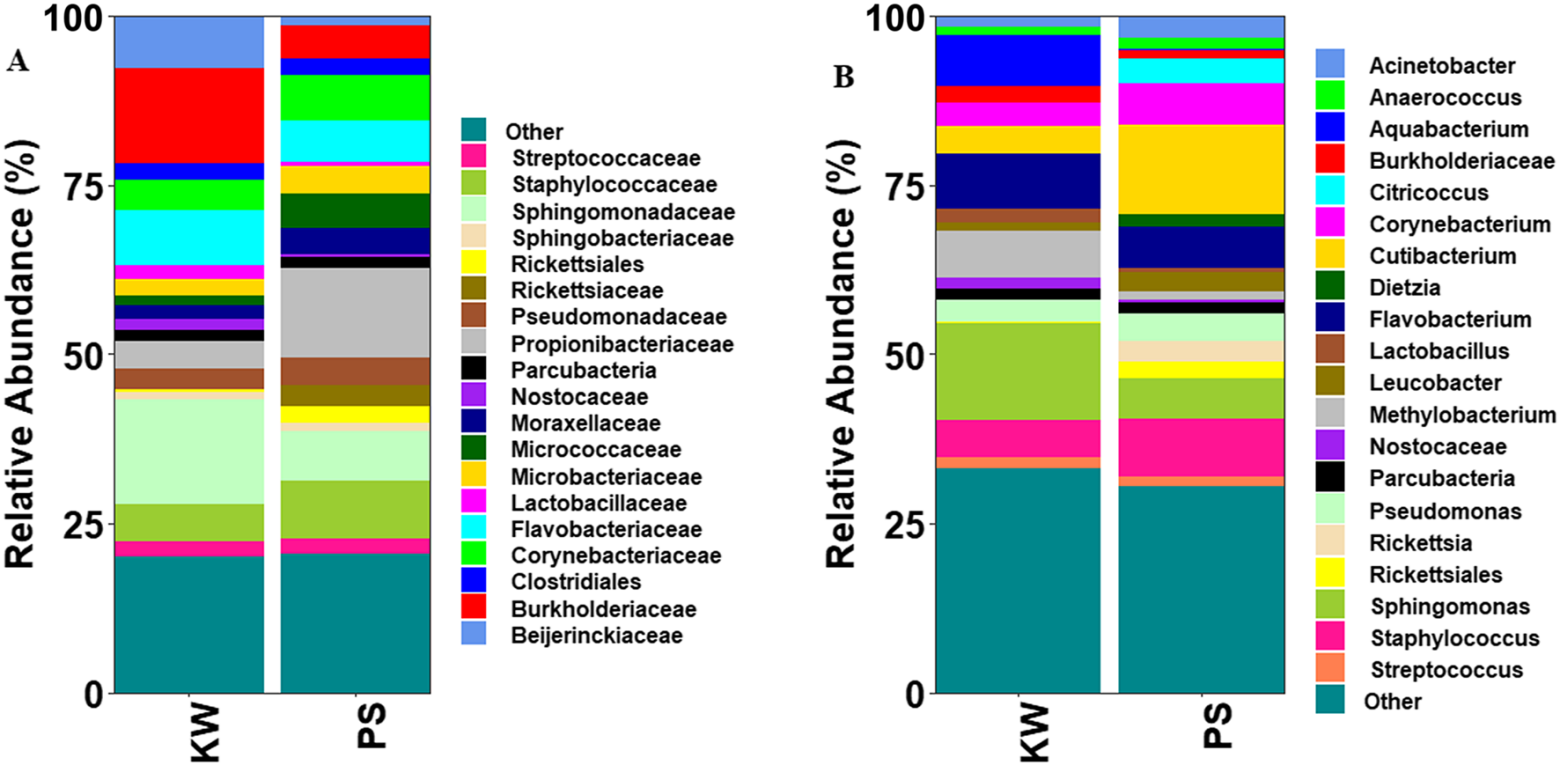
Relative abundances of bacterial taxa associated with permethrin-selected (PS) and parent strain (KW) of *Aedes aegypti* at (**A**) family and (**B**) genus level.

Proteobacteria comprising of Alphaproteobacteria (21.75%), Betaproteobacteria (10.42%) and Gammaproteobacteria (9.11%) was the most abundant bacterial phylum accounting for 41.53% of the total sequences (Fig. 1). Other common bacterial phyla identified included Actinobacteria (26.19%), Firmicutes (15.13%), Bacteroidetes (10.31%), Cyanobacteria (2.14%) and Pestescibacteria (2.04%). Actinobacteria and Gammaproteobacteria were more abundant in PS population compared to KW population while Alphaproteobacteria, Betaproteobacteria and cyanobacteria were more abundant in KW population compared to PS population. The 10 most abundant families were Sphingomonadaceae, Burkholderiaceae, Propionibacteriaceae, Flavobacteriaceae, Staphylococcaceae, Corynebacteriaceae, Beijerinckiaceae, Pseudomonadaceae, Microbacteriaceae, and Micrococcaceae and accounted for 63.91% of the total sequences (Fig. 2). Sphingomonadaceae, Burkholderiaceae and Beijerinckiaceae were more abundant in KW strain compared to PS strain while Propionibacteriaceae, Micrococcaceae, Rickettsiaceae, and unclassified Rickettsiales were more abundant in PS strain compared to KW strain. The remaining families had similar abundances between the two strains.

At the genus level, *Sphingomonas* spp., *Cutibacterium* spp., *Flavobacterium* spp., *Staphylococcus* spp., *Corynebacterium* spp., *Methylobacterium* spp., *Aquabacterium* spp., *Pseudomonas* spp., *Acinetobacter* spp. and *Leucobacter* spp. were the 10 most abundant genera and accounted for 53.82% of the total sequences (Fig. 2). *Sphingomonas* spp, *Aquabacterium* spp. and *Methylobacterium* spp. were more abundant in KW strain compared to PS strain. In contrast, *Cutibacterium* spp., *Leucobacter* spp., *Rickettsia* spp. and unclassified Rickettsiales were more abundant in PS strain compared to KS strain.

**Figure 3:**
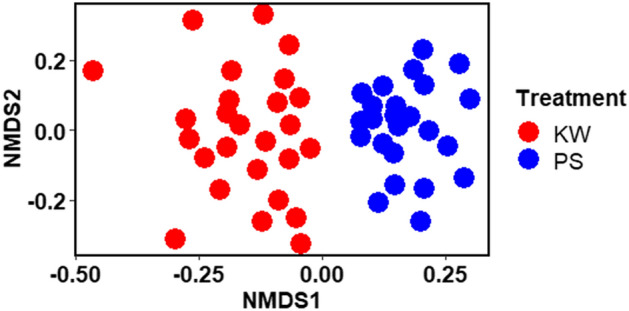
Non-metric multidimensional scaling (NMDS) ordination of Bray–Curtis distances between bacterial communities from permethrin-selected (PS) and parent strain (KW) of *Aedes aegypti*.

NMDS ordination was used to compare community membership of gut microbial communities between KW and PS strains (Fig. 3). The results indicated a clear separation between the KW and PS samples. The stress value was 0.197 indicating accurate representation of the relationship between these samples. ANOSIM analysis confirmed that bacterial communities differed significantly between the two strains (Global R = 0.590, *P* < 0.0001).

SIMPER analysis was conducted at the genus level to determine the likely drivers of differences in microbial communities between KW and PS strains. The overall average dissimilarity between KW and PS strains was 69.78. Fourteen bacterial genera contributed more than 1% to the dissimilarity between KW and PS strains (Table 1). *Cutibacterium* spp., *Corynebacterium* spp., *Citricoccus* spp., *Leucobacter* spp., *Acinetobacter* spp., *Dietzia* spp., and *Anaerococcus* spp. were more abundant in PS strain than in KW strain and accounted for 28.67% of the total average dissimilarity between the two strains. Conversely, *Aquabacterium* spp., *Methylobacterium* spp., *Flavobacterium* spp., *Lactobacillus* spp., unclassified Burkholderiaceae and unclassified Nostocaceae were more abundant in KW strain compared to PS strain and contributed 27.63% of the total average dissimilarity between groups.

**Table 1:** SIMPER analysis results comparing PS and KW samples.

Taxon	Average dissimilarity^a^	Contribution^b^ %	Cumulative %	Mean abundance KW	Mean abundance PS
*Cutibacterium*	7.372	10.57	10.57	3.92	13.20
*Aquabacterium*	5.966	8.551	19.12	7.61	0.184
*Methylobacterium*	4.584	6.57	25.69	6.79	1.19
*Flavobacterium*	4.096	5.871	31.56	8.22	6.13
*Corynebacterium*	3.041	4.359	35.92	3.49	6.17
*Citricoccus*	2.641	3.785	39.70	0.0315	3.57
*Leucobacter*	2.434	3.489	43.19	1.26	2.95
*Acinetobacter*	1.824	2.614	45.80	1.76	3.29
*Lactobacillus*	1.647	2.36	48.16	2.0	0.628
Burkholderiaceae	1.551	2.223	50.39	2.35	1.30
Parcubacteria	1.538	2.204	52.59	1.71	1.60
Nostocaceae	1.432	2.052	54.64	1.66	0.282
*Dietzia*	1.396	2.001	56.64	0.0973	1.82
*Anaerococcus*	1.298	1.86	58.50	1.16	1.59

The Bray–Curtis dissimilarity between PS and KS was greater than 1% for these 14 microbial genera.

^a^Bray–Curtis average dissimilarity between PS and KS populations, expressed as a percentage.

^b^Contribution to dissimilarity between PS and KS population. KW, parent population, PS, permethrin-selected population.

### Bacterial diversity and richness

Mean (± SE) OTU richness (observed OTUs) in PS population was 96.0 ± 3.59 and significantly higher than 84.77 ± 3.78 in parental KW population (Kruskal–Wallis chi-square = 4.24, df = 1, *P* = 0.039). Shannon diversity index in PS population was 3.59 ± 0.08 and was not significantly different from 3.43 ± 0.08 for the parental KW (Kruskal–Wallis chi-square = 2.85, df = 1, *P* = 0.09).

### Putative functional differences between bacterial microbiota of KW and PS mosquitoes

**Figure 4:**
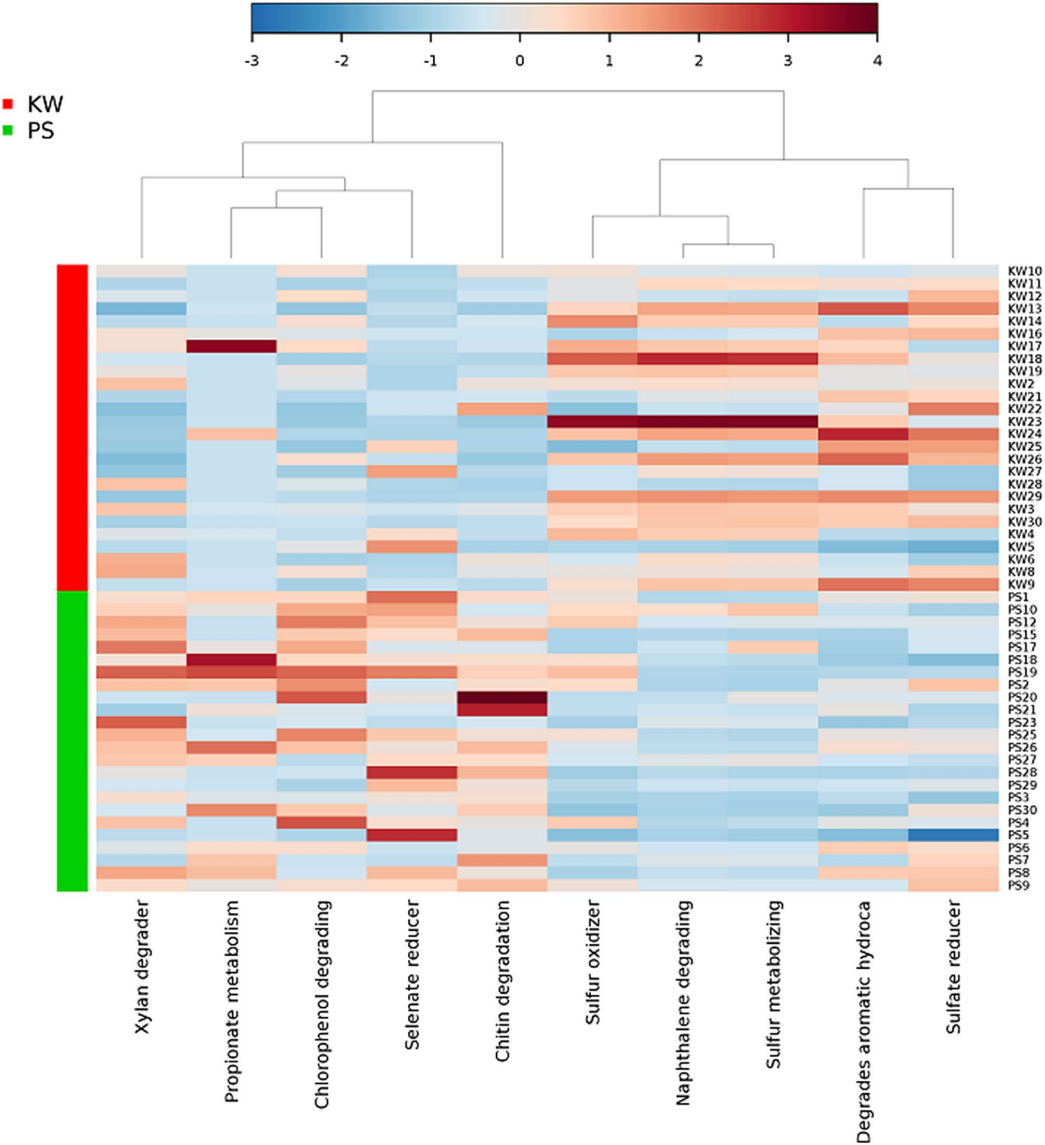
Taxonomic-based functional profiling of bacterial communities from permethrin-selected (PS) and parent strain (KW) of *Aedes aegypti*. Functional differences are shown on a relative scale with enrichment in red and depletion in blue.

Taxonomy-based functional profiling revealed differentially enriched microbial processes between the two mosquito strains (Fig. 4). KW samples were represented by an enrichment of bacteria capable of processes such as sulfur oxidation, sulfur metabolism, sulfate reduction, and degradation of complex organics such as naphthalene and aromatic hydrocarbons. In contrast, enriched processes in PS samples included propionate metabolism, selenate reduction, and degradation of xylan, chitin and chlorophenol.

## Discussion

Unraveling the composition and diversity of microbial communities that inhabit the guts of disease vectors is a critical step towards more focused studies on their contribution to host processes. This study investigated the gut bacterial communities of permethrin-selected (PS) and parental field-collected (KW) strains of the yellow fever mosquito, *Ae. aegypti*. Permethrin-selected strain had significantly higher gut microbial richness compared to the parental KW strain. The microbial composition also differed significantly between the two strains, with *Sphingomonas*, *Aquabacterium* spp. and *Methylobacterium* spp. being more abundant in KW strain compared to PS strain and *Cutibacterium* spp., *Leucobacter* spp., *Rickettsia* spp. and unclassified Rickettsiales being more abundant in PS strain compared to KS strain.

Differences in microbial communities between insecticide-resistant and -susceptible strains of insects have been reported before^8,12,20^. Chlorpyrifos- and fipronil-resistant strains of *P. xylostella* (L.) had a higher proportion of Lactobacillales and a lower proportion of Enterobacteriaceae in their midguts compared to the susceptible strain^12^. Deltamethrin-resistant population of *P. xylostella* had a lower proportion of Cyanobacteria compared to the susceptible strain, but there were no differences in microbial diversity between resistant and susceptible strains^20^. However, field-collected populations of *P. xylostella* had different microbial composition and higher microbial diversity compared to both deltamethrin-susceptible and -resistant strains^20^. In *An. albimanus* mosquito, the microbial composition and diversity differed between fenitrothion-susceptible and -resistant strains, with the susceptible strain having significantly higher microbial diversity compared to the resistant strain^8^. Additionally, *An. arabiensis* strain that was resistant to DDT, permethrin, deltamethrin, lambda-cyhalothrin and malathion had a lower microbial diversity compared to the susceptible strain^21^. *Aedes aegypti* larvae that were most tolerant to the microbial larvicide, *Bacillus thuringiensis israelensis* had significantly lower microbial richness and diversity compared to the least tolerant larvae^22^. Therefore, our findings of higher OTU richness in permethrin-selected strain relative to the parental strain is inconsistent with previous studies.

It is unclear why permethrin-selected strain was associated with higher microbial richness relative to the parental strain, but we can offer some potential hypotheses. First, exposure of permethrin-selected strain to permethrin may have reduced (not eliminated) the abundance of competitively superior but permethrin-sensitive bacterial taxa, releasing the competitively inferior but permethrin-tolerant bacterial taxa from interspecific competition and thus allowing them to proliferate. Some bacteria are also known to degrade pesticides and utilize them as a carbon source and may also have benefitted from the reduced growth of pesticide-sensitive bacterial species^23^. Previous studies have also reported significant upregulation of detoxification genes such as cytochrome P450s and Glutathione S transferases in insecticide-resistant strain of *Ae. aegypti* compared to the susceptible strain^24^. These changes may modify the gut conditions in ways that may render the mosquito more or less efficient at controlling the densities of different bacterial taxa allowing some otherwise rare bacterial taxa to thrive. For example, insecticide-resistant strain of the northern house mosquito, *Cx. pipiens* have been shown to be less efficient at controlling *Wolbachia* spp. relative to the susceptible strain^25^. Conversely, the presence of higher *Rickettsia* densities in sweet potato whitefly, *B. tabaci* was associated with higher susceptibility to insecticides^14,15^. We found higher abundance of *Rickettsia* spp. in PS strain relative to the parent strain suggesting that this bacterial genus does not enhance the susceptibility of *Ae. aegypti* to permethrin. The low abundance of some bacterial taxa such as *Sphingomonas*, *Aquabacterium* spp. and *Methylobacterium* spp. in permethrin-selected strain relative to the parent strain seems to suggest that their growth was either slowed by exposure to permethrin or that permethrin-selected strain was better able to control their densities. It is also possible that the impact of insecticide-resistance on microbial composition and richness may vary by mosquito species, type of insecticide, and the underlying insecticide resistance mechanism(s). For example, in *An. albimanus*, the microbial composition differed between fenitrothion-susceptible and -resistant strains, but not between pyrethroid-susceptible and -resistant strains^7,8^. Additional studies incorporating microbial characterization and transcriptome profiling of microbial and mosquito genes in relation to different mechanisms of insect resistance will be more revealing.

Fourteen bacterial genera accounted for 58.5% of the observed differences between permethrin-selected strain and the parent strain. It is unclear whether differential abundance of these bacterial genera between the two mosquito strains was due to their varying ability to degrade permethrin and utilize it as a source of carbon or strain-specific differences in modulation of immune system leading to differential effects on gut microbial taxa. However, some of the bacterial genera that were differentially abundant in permethrin-selected population are known to degrade insecticides. For example, *Corynebacterium* spp. has been shown to degrade carbaryl insecticide^26^ while *Acinetobacter* spp. is known to degrade cypermethrin^27^.

Taxonomic to phenotypic mapping of functional profiles of gut bacterial communities revealed that various biogeochemical and biodegradation activities occur in the gut of *Ae. aegypti* with the two mosquito strains exhibiting distinctive differences in metabolic composition. The parent strain was enriched with bacteria involved in sulfur cycling and degradation of aromatic hydrocarbons. Sulfur cycling bacteria are commonly reported both in mosquito guts and in their larval habitats. Aromatic compounds such as naphthalene are abundant and widespread environmental pollutants in freshwater bodies particularly in urban and oil spillage areas^28,29^. These compounds serve as growth substrates for microorganisms^30^ and mosquito larvae are often exposed to these compounds. Enrichment of bacteria involved in degradation of these compounds is therefore likely to be an adaptive response against exposure to these compounds in nature. Conversely, the gut of PS strain was characterized by enrichment of propionate metabolizers, selenate reducers and xylan, chlorophenol, and chitin degraders. Xylan is a hemicellulose found in plants and xylan degraders may facilitate digestion of this recalcitrant diet. Chitin is a structural part of the insect cuticle, peritrophic matrix, salivary gland, trachea, eggshell and muscle attachment points and protects the insect from mechanical and chemical damage and infection from microorganisms^31^. Enrichment of chitin degraders in PS strain may therefore impose a fitness cost on the mosquito if it results in destruction of chitin-containing structural components of the host. Further studies are needed to determine the relationship between enrichment of chitin degraders and fitness cost in PS strain. Chlorophenol compounds are ubiquitous pollutants released into the environment via industrial wastes and degradation of various pesticides^32,33^. Enrichment of bacteria with ability to metabolize these compounds in permethrin-selected population may have played a role in detoxification of permethrin and other compounds ingested by the mosquito. When exposed to pesticides, microbes are known to produce detoxification enzymes that degrade pesticides and assist the host in metabolic detoxification^34,35^.

Given the importance of mosquitoes as vectors of devastating and life-threatening diseases of public, veterinary and wildlife significance, and the persistent problem of mosquito resistance to insecticides, the potential for mosquito-associated microbiota to mediate insecticide resistance requires further investigations. These studies should establish whether the observed differences in microbial communities between the permethrin-selected strain and the unexposed parent strain are an indication of these microbes being victims of insecticide exposure or a reflection of their potential role in mediating insecticide resistance. Our results present a starting point for more focused hypothesis-driven studies to establish the relationship between gut microbiota and insecticide resistance in mosquitoes. These studies may propel the development of novel approaches for managing insecticide resistance in mosquitoes and other arthropod vectors through manipulation of gut microbes. This could be achieved by disrupting the microbial strains contributing to insecticide resistance or replacing the gut flora of resistant mosquito populations with those of susceptible populations through sugar feeding or other methods.

## Methods

### Mosquitoes and experimental design

*Aedes aegypti* mosquito larvae were collected from container habitats in Key West, Florida (KW strain) and maintained under standard insectary conditions (26 °C, 14:10 h light/dark period and 70% relative humidity). To establish a permethrin resistant strain, male and female mosquitoes were separated at the pupal stage and adults of the two sexes were separately exposed to permethrin using the standard bottle bioassay^36^ with slight modifications. In brief, up to sixty 3–4 days old adults were placed in 1L glass bottles coated with permethrin (up to 60 µg) for 60–90 min. Approximately 400 adult mosquitoes surviving permethrin exposure were mass-mated, blood-fed and used to establish successive generations. The same procedure has been repeated every generation increasing permethrin concentration to achieve 30–40% mortality. The F22 generation of both permethrin-selected (PS) and KW populations were used for this study. Larvae of both mosquito populations were reared in plastic trays in batches of 200 individuals per tray and fed on a mixture of liver powder and brewer’s yeast (1:1) for microbiome study.

### DNA extraction, library preparation and sequencing

Thirty newly emerged adult females of each mosquito population were cold anaesthetized, and their midguts dissected under a stereo microscope. Before midgut dissections, adult females were surface-sterilized by rinsing them once in 2% bleach solution for 10 min, twice in 1× sterile phosphate buffered saline (PBS) for 5 min, once in 70% ethanol for 5 min, and twice in sterile ultrapure distilled water (Invitrogen, Grand Island NY). Individual midgut samples were dissected on a drop of PBS and transferred into sterile microcentrifuge tubes containing 100 µL of sterile PBS. DNeasy Blood and Tissue Kit (Qiagen, Valencia, CA) was used to isolate DNA from individual midguts. DNA was eluted to a final volume of 100 µL and stored at − 20 °C. Primers 341f and 806r were used to amplify the V3-V4 variable region of the 16S rRNA gene^37,38^. DNA libraries were prepared and sequenced as described in Muturi et al.^39^. Sequencing was performed on an Illumina MiSeq system using the MiSeq V3 2 × 300 paired-end sequencing kit. Demultiplexed reads were examined using CLC genomics workbench v8.5 (Qiagen inc., Valencia, CA) and low-quality reads and chimeras were removed. The reads were then paired, trimmed to fixed length, and CLC Bio Microbial Genomics module was used for operational taxonomic unit (OTU) clustering. The SILVA rRNA gene database was used to assign OTUs at 97% sequence similarity^40^.

### Statistical analyses

Statistical analyses were conducted using R version 3.3.2 (https://cran.r-project.org/bin/windows/base/old/3.2.3/), PAST version 4.03 and METAGENassist analytical pipeline^41,42^. Before downstream analyses, OTUs with less than 10 sequences were discarded to reduce the problem of spurious OTUs^43^. Ten samples with less than 1218 sequences (4 and 6 KW and PS samples, respectively) were also discarded. Because alpha diversity is sensitive to uneven sampling depths across samples, all samples were rarefied to an even depth of 1218 before its estimation. Alpha diversity metrics including Shannon diversity index and observed OTUs (richness) were computed using *vegan* package in R^44^ and non-parametric Kruskal–Wallis test was used for statistical comparisons between the two mosquito strains. Non-metric multidimensional scaling (NMDS) with Bray–Curtis similarity matrix values was conducted using *vegan* package. Non-rarefied data was transformed into proportion before analysis and *Phyloseq* package was used to generate NMDS plots to visualize within-group and between-group differences in bacterial communities between mosquito strains. Stress values were used to assess whether the resulting dimension adequately represented the complete multidimensional space with stress values < 0.2 considered to be a good representation of the data^45^. Non-parametric permutation analysis of similarity (ANOSIM) test was conducted using *vegan* package to determine statistical significance. Bray–Curtis similarity percentages (SIMPER) analysis was used to determine the microbial genera driving dissimilarity of microbial communities between the two mosquito populations.

Taxonomic-to-phenotypic mapping to assess the putative functional profiles of the detected microbiota was conducted using METAGENassist analytical pipeline^41^. Taxonomic abundance data was normalized over sample by sum and over taxa by Pareto scaling and then analyzed for “metabolism by phenotype”. The results were used to generate a heatmap comparing the putative functional profiles of KW and PS mosquito strains.

## Data Availability

All relevant data are either within the paper or will be submitted in a public repository at NCBI upon acceptance of the manuscript.
